# Improvement in Glucometric Outcomes After Control-IQ Initiation in Pediatric and Adolescent Type 1 Diabetes Patients: The Impact of Basal Time in Range

**DOI:** 10.3390/ijms26199638

**Published:** 2025-10-02

**Authors:** Ana Gómez-Perea, Alfonso Lendínez-Jurado, Silvia Gallego-Gutiérrez, Fuensanta Guerrero-Del-Cueto, Ana García-Ruiz, Cristina López-De La Torre, Fernando Cardona-Díaz, Isabel Leiva-Gea

**Affiliations:** 1 Department of Pediatric Endocrinology, Regional University Hospital of Málaga, 29011 Málaga, Spain; gomezpereana@gmail.com (A.G.-P.); sgallegog@hotmail.com (S.G.-G.); isabeleivag@gmail.com (I.L.-G.); 2 Instituto de Investigación Biomédica de Málaga (IBIMA)-Plataforma BIONAND, 29010 Málaga, Spainfernando.cardona@uma.es (F.C.-D.); 3 Department of Pharmacology and Pediatrics, Faculty of Medicine, University of Malaga, Andalucía Tech, Campus de Teatinos s/n, 29071 Málaga, Spain; 4 Distrito Sanitario Málaga-Guadalhorce, 29009 Málaga, Spain; anagaruu@gmail.com; 5 Department of Biomedicine and Dentistry, Faculty of Biomedical Sciences and Sports, Universidad Europea de Andalucía, 29010 Málaga, Spain; cristina.lopez3@universidadeuropea.es; 6 Department of Surgical Specialties, Biochemistry, and Immunology, Faculty of Medicine, University of Malaga, Andalucía Tech, Campus de Teatinos s/n, 29071 Málaga, Spain

**Keywords:** pediatric type 1 diabetes, advanced hybrid closed-loop, time in range, time in hyperglycemia, time in hypoglycemia, Tandem Control-IQ

## Abstract

The development of closed-loop systems represents an evolutionary advance in the management of patients with type 1 diabetes (T1D). This study aimed to analyze the impact of the Control-IQ advanced hybrid closed-loop (AHCL) system on glucometric outcomes in a pediatric and adolescent population with T1D, comparing results with baseline values and assessing the influence of baseline Time in Range (TIR) on glycemic control in children under 6 years old over a 12-month period. A 12-month prospective analysis was conducted in 26 patients with T1D (aged 2–15 years) initiating the Control-IQ system. Glucometric variables were assessed at baseline (before system implementation) and at 1, 3, 6, and 12 months post-implementation. A subgroup analysis was performed in patients under 6 years old (*n* = 13), to evaluate the relationship between basal TIR and glucometric outcomes during the follow-up. TIR increased significantly from 62.04% at baseline to 72.50% at one month (from 57.58% to 66.18% in patients under 6 years), with this improvement sustained throughout follow-up. Time in hyperglycemia 180–250 mg/dL (TAR1) also showed significant improvement (26.84% to 17.40% at one month; 28.66% to 20.09% in patients under 6 years), with significant reductions maintained at all timepoints. Stratification according to the proportion of patients meeting consensus targets revealed significant improvements in TIR and TAR2 at 1 and 12 months in the overall cohort, though not in the under-6 subgroup. Significant differences in TIR and coefficient of variation (CV) were observed based on baseline TIR categorization (<70% vs. ≥70%). Our study revealed a significant enhancement in TIR and time spent in hyperglycemia from the first month after the implementation of the closed-loop system, which was maintained at 12 months, in both the overall cohort and the subgroup under 6 years old. In this younger subgroup, baseline TIR predicted subsequent glycemic control, with higher baseline TIR associated with better long-term outcomes in both TIR and CV.

## 1. Introduction

Type 1 diabetes (T1D) represents the most prevalent form of diabetes in pediatric populations. This chronic multifactorial disease involves a strong genetic component interacting with environmental trigger factors [1], ultimately leading to insulin deficiency due to pancreatic β-cell destruction [2].

In healthy individuals, hypoglycemia stimulates α-cells to secrete glucagon, promoting hepatic glucose production through gluconeogenesis and glycogenolysis. This regulatory process involves both intrinsic and paracrine mechanisms. The intrinsic mechanism depends on α-cell glucose metabolism, where reduced glucose transport via GLUTs (Glucose Transporter) and SGLTs (Sodium-Glucose Linked Transporter) occurs during hypoglycemia. Paracrine control involves factors secreted by β-cells (insulin, zinc, GABA), δ-cells (somatostatin), enteroendocrine cells (incretins), and adrenal medulla cells (adrenaline) [3].

In T1D, this regulatory mechanism becomes compromised, resulting in hyperglucagonemia that exacerbates hyperglycemia. Recent studies using pancreatic islets from healthy donors, glutamic acid decarboxylase autoantibody-positive (GADA+) individuals, and T1D patients indicate that α-cell dysfunction precedes overt β-cell loss. Transcriptomic analyses associate this progressive dysfunction with downregulation of key transcription factors (including ARX, MAFB, and RFX6) and signaling pathways essential for α-cell identity in T1D. During early GADA+ stages, upstream defects involve impaired expression of genes critical for glucose sensing and cellular ATP production, suggesting intrinsic metabolic defects in α-cells represent an early pathogenic mechanism in T1D [4,5].

The incidence of T1D in Spanish children under 15 years is approximately 17.69 cases per 100,000 person-years [6], with a recognized north–south gradient ranging from 0.95 to 1.7 cases per 1000 inhabitants [7]. According to American Diabetes Association (ADA) criteria, diagnosis requires HbA1c ≥ 6.5%, fasting plasma glucose ≥ 126 mg/dL, 2-h post-load glucose ≥ 200 mg/dL, or random glucose ≥ 200 mg/dL with classic symptoms—with confirmation needed in asymptomatic cases [8].

Continuous glucose monitoring has established HbA1c < 7% as a key glycemic goal in T1D management. However, the latest ADA update recommends a more stringent target of HbA1c ≤ 6.5% for individuals with access to advanced diabetes technologies, such as CGM and automated insulin delivery systems. These guidelines also recommend maintaining a Time in Range (TIR; glucose 70–180 mg/dL) of >70%, Time Below Range Level 1 (TBR1; glucose 54–70 mg/dL) of <4%, Time Below Range Level 2 (TBR2; glucose <54 mg/dL) of <1%, Time Above Range Level 1 (TAR1; glucose 180–250 mg/dL) of <25%, and Time Above Range Level 2 (TAR2; glucose >250 mg/dL) of <5% [9].

Concurrently, algorithm-driven insulin infusion systems have been developed to mimic physiological insulin secretion. These systems administer rapid-acting insulin subcutaneously, featuring continuous basal rates adjusted according to individual needs based on interstitial glucose monitoring, plus automated bolus delivery for meals and hyperglycemia correction. Multiple studies confirm their efficacy in improving glycemic outcomes [10,11,12,13,14,15].

In addition to the many published studies on the improvement of metabolic control, there are cost-utility and cost-effectiveness studies that recognize closed-loop systems as cost-effective compared to treatment with multiple daily injection (MDI) or continuous subcutaneous insulin infusion (CSII) in conjunction with continuous glucose monitoring systems (CGM) [16,17,18]. It has also been proven that these systems are safe in situations where children’s meals, schedules, and activities deviate from their pre-programmed routines, such as diabetes camps [19,20,21].

Three hybrid closed-loop systems are approved for pediatric use in Europe: CamAPS FX (CamDiab, Cambridge, UK), Medtronic MiniMed™ 780G (Medtronic, Northridge, CA, USA), and the Tandem Control-IQ System (Tandem Diabetes Care, San Diego, CA, USA). This latter system utilizes an insulin pump (t:slim ×2 insulin pump) with Control-IQ technology, providing automatic basal adjustments and correction boluses every 5 min based on Dexcom G6 CGM data. While initially approved for ages ≥6 years [22,23], recent studies confirm its safety and efficacy in children aged 2–6 years [24,25].

Our study aimed to analyze glucometric outcomes in pediatric and adolescent patients with T1D following one year of Control-IQ advanced hybrid closed-loop system (AHCL) utilization, with particular focus on assessing baseline TIR impact in the subgroup under 6 years of age.

## 2. Results

### 2.1. Study Participants

A total of twenty-six patients were included, of whom 15 (57.69%) were male. The mean age was 7.60 years (range 2–15 years). The mean age at diabetes onset was 3.79 years (range 7 months to 10.60 years). The AHCL system was implemented at a mean age of 6.40 years (range 1.91–13.80 years). Following system initiation, the mean sensor usage was 96.32%, with the lowest usage observed at three months (94.71%). The mean time in Auto Mode was 92.01%, reaching its lowest point at twelve months (88.29%). Prior to the system change, the insulin distribution was 40.10% basal and 59.90% bolus, with no significant differences observed across the subsequent follow-up timepoints.

### 2.2. Glucometric Outcomes

Mean glucose was calculated at baseline and following AHCL system initiation. The baseline value was 163.88 mg/dL, which showed a statistically significant decrease after implementation of the AHCL system. The GMI was 7.32% with the initial system and decreased to 6.94% at the first month post-initiation. This value remained stable at all subsequent timepoints, with the differences being statistically significant at 1, 3, and 6 months. The mean daily carbohydrate intake after initiating the new system was 154.46 g/day for the first 6 months, with a non-significant increase to 170.72 g/day at 12 months (Table 1).

The baseline HbA1c was 6.82%, with no significant differences observed at subsequent timepoints. Concerning CV, the baseline value was 35.24%, and no significant differences were found during the follow-up period (Table 1).

Analysis of TIR revealed significant differences across the timepoints. The mean TIR increased from a baseline value of 62.04% to 72.50% at one month (*p* < 0.000), and this improvement was sustained throughout the follow-up, reaching 75.04% at 12 months (*p* < 0.000) (Table 1). When patients were stratified based on achieving the consensus target (TIR > 70%), the proportion of patients meeting this goal increased significantly from a baseline of 32% to 54.55% at 1 month (*p* < 0.015) and to 72% at 12 months (*p* < 0.027) (Table 2).

The evaluation of TAR1 showed a baseline value of 26.84% prior to AHCL system use. Following system initiation, the mean TAR1 decreased significantly at all timepoints, reaching a minimum of 15.20% at 6 months (*p* < 0.000) (Table 1). When patients were stratified by the consensus target (TAR1 < 25%), the proportion meeting this goal increased from a baseline of 40% to 90.91% at one month; however, this increase was not statistically significant at any timepoint (Table 2).

For TAR2, the baseline value was 8.60%, with no significant improvement at subsequent timepoints (Table 1). When stratified by the TAR2 target (<5%), 56% of patients met the target at baseline. This proportion decreased to 27.27% one month after system initiation (*p* < 0.039), followed by a progressive increase to a maximum of 48% at 6 months (*p* < 0.032) (Table 2).

No significant differences were observed in TBR1 and TBR2 across the timepoints, either in mean values or in the proportion of patients achieving the targets of TBR1 < 4% and TBR2 < 1% (Table 1 and Table 2).

Stratification of the cohort by baseline TIR (≥70% vs. <70%) revealed that the improvement in TIR was primarily driven by the subgroup that initially did not meet the consensus target. This group showed a significant increase from 53.41 ± 13.14% at baseline to 70.87 ± 8.82% at 12 months (*p* < 0.003). In contrast, the group with already-optimized TIR at baseline maintained high levels (80.37 ± 5.15% to 83.25 ± 7.72%, *p* < 0.001). Significant differences in the CV between these subgroups were observed only at baseline (30.78 ± 2.58% vs. 37.47 ± 4.70%, *p* < 0.006), with no significant difference at 12 months (32.25 ± 5.84% vs. 35.77 ± 7.10%, *p* = 0.239).

The application of the Benjamini–Hochberg correction confirmed sustained, statistically significant improvements in TIR, TAR1 and mean glucose levels across all follow-up timepoints (1, 3, 6, and 12 months). Conversely, no statistically significant differences were detected—either before or after correction—in TAR2, TBR1, TBR2, GMI, HbA1c or CV.

### 2.3. Subanalysis in Children ≤6 Years

A subgroup analysis was conducted in patients aged ≤6 years (*n* = 13), comprising 7 boys and 6 girls. The mean age at diabetes onset was 1.91 ± 1.10 years, and the mean age at system initiation was 4.35 ± 1.14 years.

The mean baseline glucose value was 172.33 mg/dL. A statistically significant decrease from baseline was observed following system implementation at 1 month (*p* = 0.015), 3 months (*p* < 0.013), 6 months (*p* < 0.011), and 12 months (*p* < 0.011) (Table 3).

The analysis of TIR showed a baseline value of 57.58%, with subsequent significant increases to 66.18% at 1 month (*p* < 0.004), 70.25% at 6 months (*p* = 0.0017), and 72% at 12 months (*p* = 0.003) (Table 3).

For TAR1, a significant reduction was observed following system implementation. The baseline TAR1 of 28.66% decreased to 16.75% at 12 months (*p* < 0.004). Regarding TAR2, the initial value was 11.16%, with no significant changes during follow-up (Table 3).

No statistically significant differences were observed in TBR1, TBR2 or CV across the evaluated timepoints (Table 3).

The proportion of patients achieving the TIR target (>70%) showed a statistically significant increase, from 25% at baseline to 50% at 12 months (*p* < 0.026). Significant changes were also observed in the proportion meeting the TAR2 target (<5%), which decreased at 6 months (50% vs. 58.33% at baseline, *p* = 0.022), and in those achieving the TBR1 target (<4%), which decreased at 6 months (83.33% vs. 91.67% at baseline, *p* = 0.026).

Following Benjamini–Hochberg adjustment for multiple comparisons, statistically significant improvements in both TIR and mean blood glucose levels persisted throughout the study duration, as evaluated at 1, 3, 6, and 12-month intervals. A significant reduction in TAR1 was also sustained at the 1, 6, and 12-month assessments. In contrast, the improvement in TAR1 noted at the 3-month timepoint failed to retain statistical significance post-correction (q = 0.082). No significant alterations were observed for the other metrics analyzed. The values for TAR2, TBR1, TBR2, GMI, HbA1c and CV remained unchanged from baseline in both the initial and corrected analyses.

### 2.4. Impact of Basal Time in Range

Stratification of participants based on baseline TIR (TIR ≥70% vs. <70%) revealed significant differences in long-term outcomes following closed-loop system implementation. The cohort that met the baseline TIR target maintained superior glucometric control both at baseline and at 12 months, demonstrating a higher mean TIR (81.66 ± 7.77% vs. 49.56 ± 12.09% at baseline, *p* < 0.002; 85.00 ± 11.14% vs. 66.63 ± 8.77% at 12 months, *p* < 0.018). Glycemic variability, measured by CV, also differed significantly between groups. The group that met the baseline target exhibited a lower CV both at baseline (30.43 ± 3.37% vs. 38.12 ± 3.79%, *p* < 0.021) and at 12 months (29.73 ± 5.37% vs. 40.60 ± 4.91%, *p* < 0.011).

In the subgroup of children aged ≤6 years, a directly proportional relationship was observed between the baseline TIR percentage and the TIR percentage at 12 months (R^2^ = 0.573, *p* < 0.005). A similar relationship was found between the baseline CV percentage and the CV percentage at 12 months (R^2^ = 0.4397, *p* < 0.044).

## 3. Discussion

Our results are consistent with those reported by Wadwa RP et al. [25], who evaluated the Control-IQ system in pediatric patients aged 2–6 years, comparing them to a standard-care group using either an insulin pump or multiple daily injections combined with continuous glucose monitoring. Their study included 102 patients (68 in the closed-loop group) over a 13-week follow-up period. A significant increase in TIR was observed in the Control-IQ group (from 56.70 ± 18.00% to 69.30 ± 11.10%) compared to the standard-care group (from 54.90 ± 14.70% to 55.90 ± 12.60%). The Control-IQ group also demonstrated greater improvements in hyperglycemia and mean glucose levels [25]. Similarly to our findings, no significant differences in time spent in hypoglycemia were reported, as baseline values already met consensus targets. However, in contrast to our study, their trial reported two episodes of severe hypoglycemia in the closed-loop group and one in the standard-care group, along with one case of diabetic ketoacidosis in the closed-loop group [10].

In a retrospective study by Forlenza GP et al. [27], one-year outcomes were analyzed in a sample of 5575 patients of different age groups (including 394 patients aged 6–13 years) with either T1D (89%) or T2D (11%). The authors reported a reduction in HbA1c levels and an increase in TIR across all groups. However, the improvement in TIR was more modest than that observed in our sample (60% vs. 75% in our study). Regarding time in hypoglycemia, and consistent with our findings, values were already below ADA recommendations at baseline and remained stable throughout follow-up. When analyzing the proportion of patients achieving the TIR > 70% target, the reported figures were notably low (only 11% at baseline), reaching a maximum of 22.30% by the end of the study. For time in hypoglycemia, 92% of patients met the target at baseline, a rate that remained largely unchanged (93%) during follow-up. Similarly to our study, these results indicate that a high percentage of patients met hypoglycemia goals, but a high percentage of time spent in hyperglycemia resulted in the overall lower TIR [27].

A study by Kanapka LG et al. evaluated the safety and effectiveness of the Control-IQ system in 101 children aged 6–13 years with T1D over 28 weeks [28]. This study was an extension of a 16-week randomized clinical trial (RCT) that had demonstrated a clear benefit in TIR with the Control-IQ system compared to sensor-augmented pump (SAP) therapy [22]. Based on these results, the follow-up was extended by 12 weeks to compare outcomes between two cohorts: patients who switched from SAP to the closed-loop system (SAP-CLC cohort) and those who continued using it (CLC-CLC cohort). The results showed a significant TIR improvement in the SAP-CLC cohort (from 55 ± 13% with SAP to 65 ± 10% with CLC, *p* < 0.001), along with an increase in the proportion of patients achieving the TIR target (from 14% to 36%, *p* = 0.03), observable from the first day of system implementation. In the CLC-CLC cohort, the TIR benefit was maintained throughout the 12-week extension period. The authors concluded that the closed-loop system safely improves glycemic control in children from the first day of use, with sustained benefits over 28 weeks [22,28]. In our study, we similarly observed a significant and sustained increase in TIR throughout the 12-month follow-up. Furthermore, the proportion of patients achieving the TIR > 70% goal after initiating the Control-IQ system was substantially higher than that reported by Kanapka et al., reaching 82.60% after one year [28]. 

The prospective study by Mingorance et al. [29], conducted in 71 patients with T1D aged 6–18 years, evaluated the impact of transitioning from a predictive low glucose suspend (PLGS) system to the AHCL Control-IQ system. Their findings are consistent with ours, demonstrating an increase in TIR and a reduction in time spent in hyperglycemia from the first month after system implementation, with these improvements remaining stable throughout the 12-month follow-up. In contrast to our results, a decrease in time spent in hypoglycemia was also observed in their study. Furthermore, primary caregivers reported an improvement in quality of life, attributed to better glycemic control and reduced need for nocturnal glucose monitoring—a variable that was not assessed in our study [29].

Our findings demonstrate that advanced hybrid closed-loop therapy (Control-IQ system) significantly improves glucometric parameters in children under six years with T1D, showing a 14.42 percentage-point increase in baseline TIR after 12 months of use, along with progressive reductions in TAR1 and TAR2. These results are consistent with recent literature, reinforcing the system’s efficacy for optimizing metabolic control in this particularly challenging pediatric group.

The randomized clinical trial by Ware et al. [30] corroborates our observations, reporting a 12% TIR improvement in children <6 years using hybrid closed-loop systems compared to conventional therapy. However, our study reveals important heterogeneity in treatment response based on baseline patient characteristics. While some subgroups achieved substantial TIR gains, others showed more modest improvements, highlighting the need for individualized effectiveness assessments when implementing Control-IQ technology.

Significant differences in both TIR and the CV persisted after 12 months of Control-IQ use, stratified by baseline glycemic status. Patients with a baseline TIR < 70% exhibited more pronounced TIR improvements than those with a baseline TIR ≥ 70%, although absolute TIR values remained lower in the former group. This finding is consistent with Schoelwer et al. [31], who identified baseline TIR as the strongest predictor of closed-loop system performance in pediatric T1D.

Similarly, a home-use evaluation of the Tandem Control-IQ system in young children by Forlenza et al. [32] demonstrated that the achieved TIR remains influenced by pre-implantation values. Although the average TIR exceeded 70%, participants with poorer baseline glycemic control did not achieve the same glycemic levels as those with better initial control. This pattern indicates that while hybrid closed-loop systems substantially improve glucometric outcomes, their efficacy is partially constrained by the patient’s baseline metabolic status. These observations highlight the importance of individualized system adjustments and active caregiver involvement to maximize therapeutic benefits.

The impact of glucometric data derived from continuous glucose monitoring, particularly TIR, is clinically relevant for preventing and mitigating the development of metabolic complications in patients with diabetes. Higher TIR is independently associated with a lower risk of microvascular complications, such as retinopathy, nephropathy, and neuropathy, as well as a reduced incidence of hospitalizations for hypoglycemia and ketoacidosis in type 1 diabetes [33]. Furthermore, evidence indicates that low TIR and high glycemic variability are associated with an increased risk of albuminuria, retinopathy, cardiovascular disease, and mortality [34,35].

The investigation into how these advanced technologies directly influence complex molecular pathways, specifically in children and adolescents, remains profoundly limited. The vast majority of evidence linking AHCL systems to molecular biomarkers predominantly focuses on established clinical metrics such residual C-peptide secretion [36,37,38]. A significant knowledge gap remains regarding the unique molecular response to the specific glycemic patterns achieved by AHCL systems in the developing child, whose pathophysiology and hormonal environment differ substantially from those of adults.

Several limitations of our study should be considered when interpreting the results and assessing their clinical applicability. The sample size was relatively small, particularly in the subgroup of children aged ≤6 years. Furthermore, crucial patient-reported outcomes such as quality of life and caregiver burden, which are key for evaluating the impact of AHCL systems in pediatric populations, were not assessed.

The absence of weight data precluded the calculation of insulin dosage in IU/kg/day. Additionally, a partial loss of baseline glucometric data occurred due to the deletion of information from the LibreView platform prior to closed-loop system initiation; however, these data were recovered via the digital medical record system (Diraya platform). Future studies with larger sample sizes are needed to address these limitations, alongside the development of strategies to prevent data loss. Comparing these results with those from studies utilizing different glucose management assessment methods could also provide a more comprehensive understanding of closed-loop system efficacy in this population.

A key strength of this study is the high adherence to the Control-IQ system, reflected in the high sensor usage and substantial time spent in auto mode. The therapy demonstrated a strong safety profile, with no episodes of ketoacidosis or level 3 hypoglycemia reported. The application of the Benjamini–Hochberg correction confirmed that the robust improvements in glycemic control were both sustained and statistically significant. Key metrics demonstrating this effect included a sustained increase in TIR, a reduction in mean glucose, and beneficial effects on TAR1 at most timepoints. Finally, it is important to emphasize that this study was conducted in a routine clinical practice setting rather than a controlled trial, enhancing the generalizability of the results. These findings, while limited by the sample size, provide valuable real-world evidence for the use of advanced hybrid closed-loop systems in very young children—a demographic that remains critically underrepresented in the scientific literature.

## 4. Materials and Methods

This retrospective, single-center study was conducted at the diabetes unit of a tertiary hospital in Spain (Regional University Hospital of Malaga). Data were extracted from digital medical records over a 12-month follow-up period. The study included pediatric and adolescent patients (aged 2–15 years) with a diagnosis of T1D who were transitioning from combined therapy with CSII (t:slim X2 Basal-IQ insulin pump) (Tandem Diabetes Care, San Diego, CA, USA) and a Dexcom G6 (Dexcom, Inc., San Diego, CA, USA) isCGM (intermittently scanned continuous glucose monitoring) system to the Control-IQ AHCL system with the Dexcom G6 sensor. All patients were managed by a multidisciplinary team of pediatric endocrinologists and diabetes specialist nurses.

Metabolic control variables were obtained from the LibreView^®^ (Abbott Diabetes Care, Alameda, CA, USA) and Glooko^®^ (Glooko, Inc., Palo Alto, CA, USA) download platforms at baseline and at 1, 3, 6, and 12 months. Baseline data included sensor and insulin pump readings from the two weeks preceding system upgrade. After initiation of the closed-loop system, data corresponding to the two weeks prior to each follow-up visit were downloaded using the Glooko^®^ platform.

The variables studied were:

*TIR: percentage of time in which interstitial blood glucose levels are between 70 and 180 mg/dL.
*TAR1: percentage of time in which interstitial blood glucose levels are between 180 and 250 mg/dL.
*TAR2: percentage of time in which interstitial blood glucose is above 250 mg/dL.
*TBR1: percentage of time in which interstitial blood glucose levels are between 70 and 54 mg/dL.
*TBR2: percentage of time in which interstitial blood glucose is below 54 mg/dL.

Additionally, we calculated the percentage of patients achieving consensus targets. Other parameters included were the percentage of time in auto mode, coefficient of variation (CV), percentage of insulin in basal or bolus form, the amount of insulin administered as automatic correction, Hb1Ac, carbohydrates, Glucose Management Indicator (GMI), and mean glucose.

A subgroup analysis was conducted for patients aged ≤6 years, encompassing all study variables. This cohort was further stratified based on baseline Time in Range (TIR ≥ 70% vs. <70%) to evaluate its influence on subsequent glucometric outcomes.

The study was conducted in accordance with the principles of the Declaration of Helsinki. Written informed consent was obtained from all participants and their legal caregivers. The study protocol received approval from the local ethics committee center (1299-N-23, 27 July 2023).

Data analysis was performed using free R 4.0.2 software (R-CoreTeam 2020) (https://www.r-project.org/, accessed on 25 February 2025). A Shapiro–Wilk test analysis was performed to determine the normality of the study variables. Results are presented as mean ± SD values in normal distributions or as median (interquartile range [IQR]) in nonnormal distributions. A Wilcoxon signed-rank test was performed to analyze differences in the nonnormal distributions, and the paired *t* test was used in the normal distributions. Correlation analyses were performed using Pearson’s correlation coefficient in parametric variables and Spearman’s correlation coefficient in nonparametric variables. A chi-square test was used to perform a bivariate analysis. *p* < 0.05 was considered statistically significant. *p* values were adjusted using the Benjamini–Hochberg correction for multiple comparisons.

## 5. Conclusions

The implementation of the automated system resulted in significant and sustained improvements in TIR and a reduction in hyperglycemia over the 12-month study period. The therapy demonstrated a favorable safety profile, with no episodes of severe hypoglycemia (level 3) or diabetic ketoacidosis. The observed glycemic outcomes, particularly for TIR, align with those reported in randomized controlled trials of the Control-IQ system, while contributing valuable real-world clinical evidence.

In children under 6 years with T1D, we observed overall improvements in TIR and hyperglycemia, along with a significant correlation between baseline TIR and 12-month outcomes. Stratification by baseline glycemic control (<70% vs. ≥70% TIR) revealed differential responses, with distinct follow-up patterns in both TIR and the CV. These findings underscore the clinical relevance of establishing individualized glycemic targets based on pretreatment metabolic status.

This study provides robust evidence supporting the efficacy of the Control-IQ AHCL system for improving metabolic control in pediatric T1D, including the particularly vulnerable population of children under 6 years. The combination of sustained glucometric benefits, a strong safety profile, and demonstrated real-world applicability positions this technology as a significant therapeutic advancement for this challenging patient population.

## Figures and Tables

**Table 1:** Glucometric outcomes and system usability after 12 months of use of the advanced hybrid closed-loop system Control-IQ.

	Baseline	1 Month	3 Months	6 Months	12 Months
TIR (%) (Mean ± SD) ^1^	62.04 ± 16.96	72.50 ± 10.36 (*p* < 0.000)	71.28 ± 12.81 (*p* < 0.001)	74.12 ± 11.62 (*p* < 0.000)	75.04 ± 10.01 (*p* < 0.000)
TAR1 (%) (Mean ± SD) ^1^	26.84 ± 10.50	17.40 ± 6.12 (*p* < 0.000)	19.24 ± 7.41 (*p* < 0.000)	15.20 ± 7.46 (*p* < 0.000)	16.56 ± 6.78 (*p* < 0.000)
TAR2 (%) (Mean ± SD) ^1^	8.60 ± 11.56	7.45 ± 5.65 (*p* < 0.174)	7.48 ± 7.45 (*p* < 0.289)	6.32 ± 7.30 (*p* < 0.198)	5.52 ± 5.33 (*p* < 0.142)
TBR1 (%) (Median (IQR)) ^2^	2.04 (1–3)	2.12 (1–2.75) (*p* < 0.661)	1.71 (1–2) (*p* < 0.942)	2.48 (2–3) (*p* < 0.106)	2.40 (1–3) (*p* < 0.340)
TBR2 (%) (Median (IQR)) ^2^	0.48 (0–1)	0.50 (0–1) (*p* < 0.886)	0.43 (0–1) (*p* < 0.608)	1.16 (0–1) (*p* < 0.095)	0.48 (0–1) (*p* < 0.715)
Glucose (mg/dL) (Median (IQR)) ^2^	163.88 (142–183)	151.95 (142–163.75) (*p* < 0.001)	154.19 (143–166) (*p* < 0.003)	149.04 (140–155) (*p* < 0.001)	147.64 (142–154) (*p* < 0.006)
GMI (%) (Mean ± SD) ^1^	7.32 ± 0.76	6.94 ± 0.41 (*p* < 0.033)	6.98 ± 0.53 (*p* < 0.021)	6.90 ± 0.47 (*p* < 0.021)	6.84 ± 0.39 (*p* < 0.096)
HbA1c (%) (Mean ± SD) ^1^	6.82 ± 0.58	-	6.43 ± 0.77 (*p* < 0.447)	6.24 ± 0.57 (*p* < 0.182)	6.35 ± 0.54 (*p* < 0.118)
CV glucose (%) (Mean ± SD) ^1^	35.24 ± 5.18	37.68 ± 7.29 (*p* < 0.470)	34.85 ± 5.79 (*p* < 0.373)	35.39 ± 5.88 (*p* < 0.710)	34.80 ± 6.73 (*p* < 0.563)
Total basal insulin (%) (Mean ± SD) ^1^	40.10 ± 12.39	44.26 ± 10.28 (*p* < 0.686)	44.68 ± 9.84 (*p* < 0.600)	46.31 ± 10.95 (*p* < 0.122)	45.64 ± 8.15 (*p* < 0.344)
Total bolus insulin (%) (Mean ± SD) ^1^	59.90 ± 12.39	55.74 ± 10.284 (*p* < 0.686)	54.32 ± 9.84 (*p* < 0.600)	53.69 ± 10.95 (*p* < 0.122)	54.36 ± 8.15 (*p* < 0.344)
Total autocorrection insulin (%) (Mean ± SD)	-	6.16 ± 7.31	14.53 ± 18	13.61 ± 13.87	17.81 ± 22.52
Carbohydrates intake (g/day) (Mean ± SD)	-	156.15 ± 51.59	149.97 ± 51.94	150.27 ± 44.47	170.72 ± 79.64
Sensor use (%) (Median (IQR))	-	97.75 (98–99)	94.71 (97–99)	97.80 (97.50–99)	95.01 (95–99)
Time in Auto Mode (%) (Median (IQR))	-	91.47 (96–98)	93.84 (95–97)	94.43 (96–98)	88.29 (94.50–98)

^1^ Paired *t*-test, ^2^ Wilcoxon test. Values are presented as mean ± SD or median (IQR). TIR: time in range (70–180 mg/dL); TAR1: time above range >180 mg/dL; TAR2: time above range >250 mg/dL; TBR1: time below range <70 mg/dL; TBR2: time below range <54 mg/dL; CV: coefficient of variation. Data are shown at baseline and at 1, 3, 6, and 12 months of follow-up.

**Table 2:** Percentage of patients meeting ATTD 2019 consensus criteria at the different timepoints [26].

	Baseline	1 Month	3 Months	6 Months	12 Months
TIR (% patients)	32	54.55 (*p* < 0.015)	57.14 (*p* < 0.493)	72 (*p* < 0.318)	72 (*p* < 0.027)
TAR1 (% patients)	40	90.91 (*p* < 0.600)	76.19 (*p* < 0.417)	96 (*p* = 0.388)	92 (*p* < 0.212)
TAR2 (% patients)	56	27.27 (*p* < 0.039)	33.33 (*p* < 0.043)	48 (*p* < 0.032)	48 (*p* < 0.098)
TBR1 (% patients)	92	77.27 (*p* < 0.630)	90.48 (*p* < 0.852)	80 (*p* < 0.759)	80 (*p* < 0.759)
TBR2 (% patients)	92	90.91 (*p* < 0.630)	100 (*p* < 0.852)	88 (*p* < 0.759)	88 (*p* < 0.759)

Values represent the proportion of patients who achieved the recommended thresholds for each glucometric parameter at baseline and after 1, 3, 6, and 12 months of system use. The targets applied were: TIR ≥ 70% (70–180 mg/dL), TAR1 < 25% (>180 mg/dL), TAR2 < 5% (>250 mg/dL), TBR1 < 4% (<70 mg/dL), TBR2 < 1% (<54 mg/dL).

**Table 3:** Glucometric outcomes and system usability in the subgroup ≤ 6 years after 12 months of use of the advanced hybrid closed-loop system Control-IQ.

	Baseline	1 Month	3 Months	6 Months	12 Months
TIR (%) (Mean ± SD) ^1^	57.58 ± 6.96	66.18 ± 11.04 (*p* < 0.004)	63.90 ± 13.81 (*p* < 0.003)	70.25 ± 11.83 (*p* < 0.0017)	72.00 ± 18.11 (*p* < 0.003)
TAR1 (%) (Mean ± SD) ^1^	28.66 ± 12.41	20.09 ± 4.32 (*p* < 0.016)	21.90 ± 4.55 (*p* < 0.029)	17.66 ± 6.74 (*p* < 0.010)	16.75 ± 7.13 (*p* < 0.004)
TAR2 (%) (Mean ± SD) ^1^	11.16 ± 13.95	11.09 ± 5.20 (*p* < 0.594)	11.90 ± 8.41 (*p* < 0.86)	8.83 ± 9.18 (*p* < 0.54)	8.00 ± 6.49 (*p* < 0.43)
TBR1 (%) (Mean ± SD) ^1^	2.00 ± 1.41	2.10 ± 1.32 (*p* < 0.61)	2.00 ± 1.15 (*p* < 0.34)	2.58 ± 1.37 (*p* < 0.16)	2.58 ± 1.31 (*p* < 0.22)
TBR2 (%) (Mean ± SD) ^1^	0.58 ± 0.90	0.45 ± 0.68 (*p* < 0.44)	0.60 ± 0.51 (*p* < 0.34)	0.66 ± 0.65 (*p* < 0.34)	0.66 ± 0.77 (*p* < 0.44)
Glucose (mg/dL) (Mean ± SD) ^1^	172.33 ± 30.32	160.72 ± 13.71 (*p* < 0.015)	164.60 ± 20.87 (*p* < 0.013)	154.91 ± 24.88 (*p* < 0.011)	151.75 ± 19.98 (*p* < 0.011)
GMI (%) (Mean ± SD) ^1^	7.6 ± 0.68	7.14 ± 0.32 (*p* < 0.509)	7.24 ± 0.53 (*p* < 0.397)	7.08 ± 0.54 (*p* < 0.340)	6.99 ± 0.43 (*p* < 0.528)
HbA1c (%) (Mean ± SD) ^1^	6.68 ± 0.37	-	-	6.46 ± 0.24 (*p* < 0.43)	6.68 ± 0.50 (*p* < 0.76)
CV glucose (%) (Mean ± SD) ^1^	35.55 ± 5.15	40.52 ± 3.71 (*p* < 0.49)	38.88 ± 3.60 (*p* < 0.745)	37.23 ± 5.53 (*p* < 0.804)	37.80 ± 6.66 (*p* < 0.556)
Total basal insulin (%) (Mean ± SD)	41.83 ± 12.81	43.55 ± 11.84	44.50 ± 11.59	46.67 ± 12.12	46.42 ± 8.84
Total bolus insulin (%) (Mean ± SD)	58.17 ± 12.81	56.45 ± 11.84	55.50 ± 11.59	53.33 ± 12.12	53.58 ± 8.84
Total autocorrection insulin (%) (Mean ± SD)	-	3 ± 2.68	5.3 ± 7.20	6.45 ± 7.34	7.92 ± 8.80
Carbohydrates intake (g/day) (Mean ± SD)	-	141.99 ± 56.02	138.27 ± 57.21	129.13 ± 45.59	165.06 ± 93.88
Sensor use (%) (Median (IQR))	-	98.18 (98–98.5)	91.20 (96.25–98)	97.73 (97–99)	92.19 (95–98.25)
Time in Auto Mode (%) (Median (IQR))	-	93.09 (97–98)	92.50 (95.25–97.75)	97 (96.50–98)	88.92 (95.75–98)

^1^ Paired *t*-test. Values are presented as mean ± SD or median (IQR). TIR: time in range (70–180 mg/dL); TAR1: time above range >180 mg/dL; TAR2: time above range >250 mg/dL; TBR1: time below range <70 mg/dL; TBR2: time below range <54 mg/dL; CV: coefficient of variation. Data are shown at baseline and at 1, 3, 6, and 12 months of follow-up.

## Data Availability

The original contributions presented in this study are included in the article. Further inquiries can be directed to the corresponding author.

## Abbreviations

The following abbreviations are used in this manuscript:

AHCL	Advanced hybrid closed-loop
CGM	Continuous glucose monitoring
CLC	Closed-loop Control IQ
CSII	Continuous subcutaneous insulin infusion
CV	Coefficient of variation
GADA	Glutamic acid decarboxylase autoantibody
GLUT	Glucose transporter
GMI	Glucose Management Indicator
isCGM	Intermittently scanned continuous glucose monitoring
MDI	Multiple daily injections
PLGS	Predictive low glucose suspend
SAP	Sensor-augmented pump
RCT	Randomized clinical trial
SGLT	Sodium-glucose linked transporter
T1D	Type 1 diabetes
T2D	Type 2 diabetes
TIR	Time In Range
TAR1	Time Above Range level 1
TAR2	Time Above Range level 2
TBR1	Time Below Range level 1
TBR2	Time Below Range level 2
